# Sip1, a Conserved AP-1 Accessory Protein, Is Important for Golgi/Endosome Trafficking in Fission Yeast

**DOI:** 10.1371/journal.pone.0045324

**Published:** 2012-09-17

**Authors:** Yang Yu, Ayako Kita, Masako Udo, Yuta Katayama, Mami Shintani, Kwihwa Park, Kanako Hagihara, Nanae Umeda, Reiko Sugiura

**Affiliations:** Laboratory of Molecular Pharmacogenomics, School of Pharmaceutical Sciences, Kinki University, Higashi-Osaka, Japan; University of Cambridge, United Kingdom

## Abstract

We had previously identified the mutant allele of *apm1^+^* that encodes a homolog of the mammalian μ 1A subunit of the clathrin-associated adaptor protein-1 (AP-1) complex and demonstrated that the AP-1 complex plays a role in Golgi/endosome trafficking, secretion, and vacuole fusion in fission yeast. Here, we isolated a mutant allele of *its4^+^/sip1^+^*, which encodes a conserved AP-1 accessory protein. The *its4-1*/*sip1-i4* mutants and *apm1* **-**deletion cells exhibited similar phenotypes, including sensitivity to the calcineurin inhibitor FK506, Cl^−^ and valproic acid as well as various defects in Golgi/endosomal trafficking and cytokinesis. Electron micrographs of *sip1-i4* mutants revealed vacuole fragmentation and accumulation of abnormal Golgi-like structures and secretory vesicles. Overexpression of Apm1 suppressed defective membrane trafficking in *sip1-i4* mutants. The Sip1-green fluorescent protein (GFP) co-localized with Apm1-mCherry at Golgi/endosomes, and Sip1 physically interacted with each subunit of the AP-1 complex. We found that Sip1 was a Golgi/endosomal protein and the *sip1-i4* mutation affected AP-1 localization at Golgi/endosomes, thus indicating that Sip1 recruited the AP-1 complex to endosomal membranes by physically interacting with each subunit of this complex. Furthermore, Sip1 is required for the correct localization of Bgs1/Cps1, 1,3-β-D-glucan synthase to polarized growth sites. Consistently, the *sip1-i4* mutants displayed a severe sensitivity to micafungin, a potent inhibitor of 1,3-β-D-glucan synthase. Taken together, our findings reveal a role for Sip1 in the regulation of Golgi/endosome trafficking in coordination with the AP-1 complex, and identified Bgs1, required for cell wall synthesis, as the new cargo of AP-1-dependent trafficking.

## Introduction

Clathrin adaptor protein (AP) complexes play a key role in the transport of proteins by regulating the formation of transport vesicles as well as cargo selection between organelles of the post-Golgi network, namely the *trans*-Golgi network, endosomes, lysosomes, and the plasma membrane [1]. Four basic AP complexes (AP-1, AP-2, AP-3, and AP-4) have been described, and all four complexes are heterotetramers comprising the two large subunits γ/α/δ/σ and β1/β2/β3/β4, a medium-sized or μ subunit, and a small or σ subunit [2], [3]. AP-1, AP-2, and AP-3 are encoded in all eukaryotic cells examined until date, whereas AP-4 is not found in the fruit fly, nematode, and the yeasts.

Recently, accessory proteins that bind to the AP complex have been attracting increasing attention for their role in membrane trafficking as well as in signaling pathways [4], [5]. Compared with AP-2-binding proteins, less is known about AP-1 accessories with the exceptions of γ-synergin [6], Eps15 [7], epsinR [8], and p200 [9]. γ-Synergin is associated with AP-1 both in the cytosol and on *trans*-Golgi network (TGN) membranes. It binds directly to the ear domain of γ-adaptin and contains an Eps15 homology (EH) domain, although the EH domain is not part of the γ-adaptin binding site.

Eps15 (EGFR pathway substrate clone 15) was first identified as a substrate for EGF receptor tyrosine kinase [10], [11]. Eps15 is well known for its role in clathrin-coated vesicle formation in the plasma membrane through interactions with other clathrin adaptor proteins such as AP-2. In addition to its localization in the plasma membrane, Eps15 is found in the TGN in association with TGN clathrin adaptor AP-1, mediating the formation of Golgi-derived vesicles [12].

EpsinR is a highly conserved clathrin-coated vesicle enriched protein that binds to phosphatidylinositol-4-phosphate, clathrin, and the gamma appendage domain of AP-1 [13]. EpsinR plays a role in AP-1/clathrin budding events in the cell. Another AP-1-interacting protein, aftiphilin, was identified after searching databases for sequences that contained the γ-ear-binding motif [13], [14]. Aftiphilin co-elutes with two other AP-1 binding partners, p200a and γ-synergin. In general, the aftiphilin/p200/γ-synergin complex has been reported to facilitate AP-1 functions although this complex may have additional functions [15].

**Table 1:** Schizosaccharomyces pombe strains used in this study.

Strain	Genotype	Reference
HM123	*h* ^−^ *leu1-32*	Our stock
KP456	*h* ^−^ *leu1-32 ura4-D18*	Our stock
SP733	*h* ^−^ *leu1-32 sip1-i4*	This study
KP630	*h* ^−^ *leu1-32 ura4-D18 apm1::ura4^+^*	Kita et al., (2004)
SP736	*h* ^−^ *leu1-32 ura4-D18 sip1-i4*	This study
KP1754	*h* ^−^ *leu1-32 ura4-D18 nmt1 GST-sip1^+^::KanMx6*	Our stock
KP1915	*h* ^−^ *leu1-32 ura4-D18 GFP-sip1^+^::KanMx6*	Our stock
SP1041	*h* ^−^ *leu1-32 ura4-D18 GFP-sip1^+^::KanMx6 apm1::ura4^+^*	This study
SP1276	*h* ^−^ *leu1-32 ura4-D18 GFP-sip1^+^::KanMx6 apl2::ura4^+^*	This study
SP1278	*h* ^−^ *leu1-32 ura4-D18 GFP-sip1^+^::KanMx6 apl4::ura4^+^*	This study
SP1294	*h* ^−^ *leu1-32 ura4-D18 GFP-sip1^+^::KanMx6 aps1::ura4^+^*	This study
KP1807	*h* ^−^ *leu1-32 ura4-294 nmt1 GFP-sip1^+^::ura4^+^*	Our stock
SP1555	*h* ^−^ *leu1-32 ura4-D18 nmt1 GFP-sip1^+^::ura4^+^ sip1-i4*	This study
SP1428	*h* ^−^ *leu1-32 sip1-62-HA-Kan*	This study
SP1931	*h* ^−^ *leu1-32 sip1-i4 Pbgs1^+^::GFP-bgs1^+^::leu1^+^*	This study
SP1941	*h* ^−^ *leu1-32 Pbgs1^+^::GFP-bgs1^+^::leu1^+^*	This study
SP1980	*h* ^−^ *leu1-32 ura4-D18 apm1::ura4^+^ Pbgs1^+^::GFP-bgs1^+^::leu1^+^*	This study
KP427	*h* ^−^ *leu1-32 ura4-D18 apl2::ura4^+^*	Y. Ma et al., (2009)
SP132	*h* ^−^ *leu1-32 ura4-D18 apl4::ura4^+^*	This study
KP3391	*h* ^−^ *leu1-32 ura4-D18 aps1::ura4^+^*	Y Ma et al., (2009)
SP1730	*h* ^−^ *leu1-32 ura4-D18 GFP-sip1^+^::KanMx6 anp1^+^-linker-mCherry::ura4^+^*	This study
SP1732	*h* ^−^ *leu1-32 ura4-D18 nmt1 GFP-sip1^+^::ura4^+^ anp1^+^-linker-mCherry::ura4^+^*	This study
SP1966	*h* ^−^ *or ^+^ leu1-32 ura4-D18 GFP-sip1^+^::KanMx6 sec72^+^-mCherry::ura4^+^*	This study
SP1973	*h* ^−^ *or ^+^ leu1-32 ura4-D18 nmt1 GFP-sip1^+^::ura4+ sec72^+^-mCherry::ura4^+^*	This study
SP1964	*h* ^−^ *or ^+^ leu1-32 ura4-D18 GFP-sip1^+^::KanMx6 vrg4^+^-mCherry::ura4^+^*	This study
SP1965	*h* ^−^ *or ^+^ leu1-32 ura4-D18 nmt1 GFP-sip1^+^::ura4^+^ vrg4^+^-mCherry::ura4+*	This study
SP2077	*h* ^−^ *leu1-32 ura4-D18 GFP-sip1^+^::KanMx6 anp1^+^-linker-mCherry::ura4^+^ apm1::ura4^+^*	This study
SP2078	*h* ^−^ *or ^+^ leu1-32 ura4-D18 GFP-sip1^+^::KanMx6 vrg4^+^-mCherry::ura4^+^ apm1::ura4^+^*	This study
SP2079	*h* ^−^ *or ^+^ leu1-32 ura4-D18 GFP-sip1^+^::KanMx6 sec72^+^-mCherry::ura4^+^ apm1::ura4^+^*	This study

In a search for mutants sensitive to the immunosuppressive drug FK506, we previously identified a mutant allele of the *apm1* ^+^ gene that encodes a μ1 subunit of the AP-1 complex and characterized the role of Apm1 in Golgi/endosome trafficking, vacuole fusion, and secretion in fission yeast [16]. In this organism, the AP-1 complex comprises [Apl2 (β), Apl4 (γ), Apm1 (μ), and Aps1 (σ)] subunits that are all localized to endosomes and are essential for heterotetrameric complex formation [17]. A recent study on budding yeast reported an evolutionarily conserved accessory protein, Laa1p (YJL207C) [18], which shares a significant amino acid similarity with human p200, one of the AP-1 accessory proteins [15]. Laa1 interacted with the clathrin-associated adapter complex AP-1 and was important for the correct localization of the AP-1 complex in Golgi/endosomes, establishing the evolutionary conservation of the function of this protein in AP-1 mediated endosomal trafficking [18]. In 2009, Jourdain *et al* reported a fission yeast member of the p200/Laa1 family, Sip1, as an essential protein that interacted with the F-box protein Pof6, and concluded that Sip1 was an endocytic vesicle protein important for endocytosis [19]. However, the role of Sip1 as an AP-1 accessory in AP-1 mediated endosomal trafficking, and its functional interactions with other signaling pathways in fission yeast remain undetermined.

**Figure 1:**
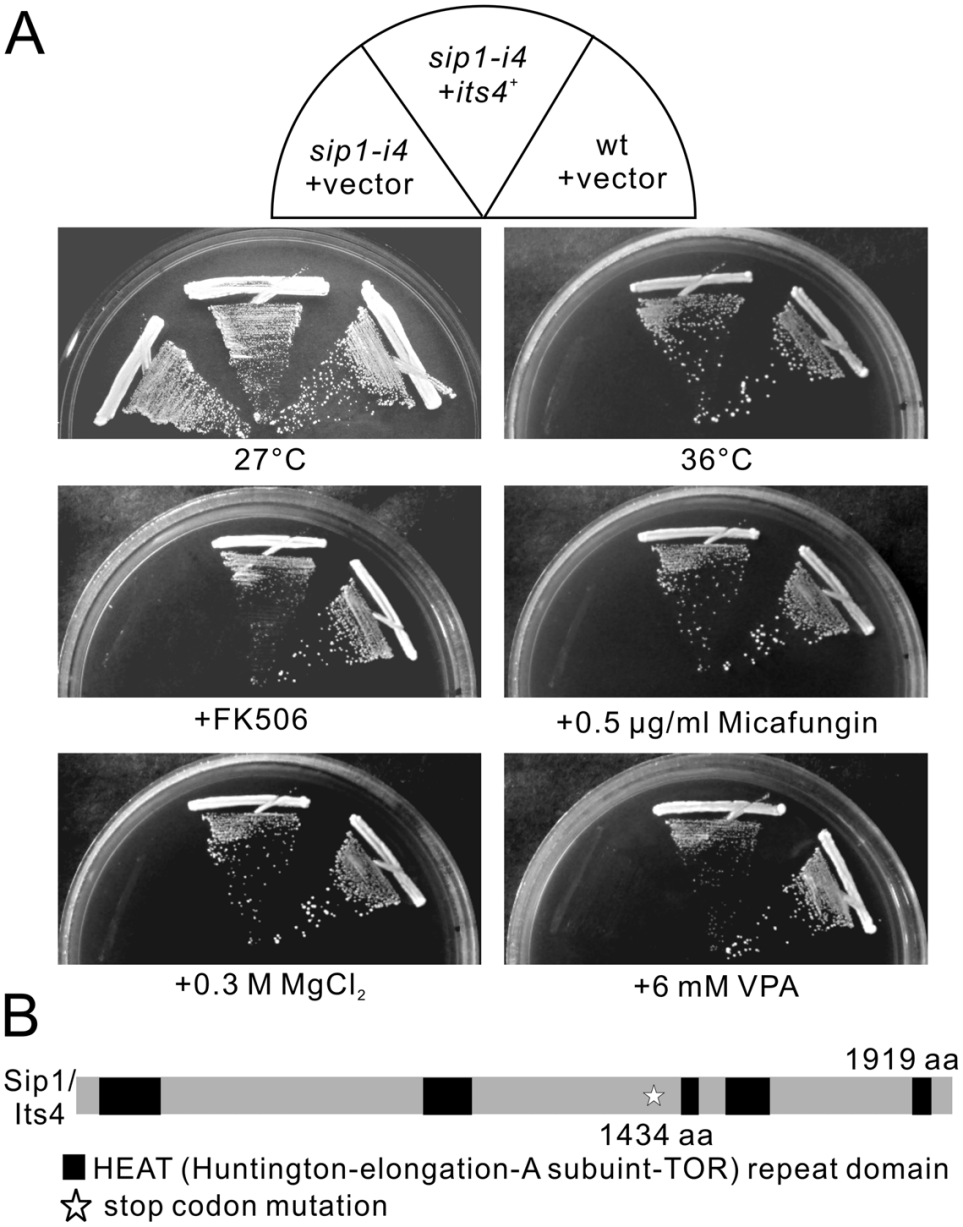
Mutation in the *sip1* ^+^ */its4* ^+^ gene caused immunosuppressant-, chloride-, valproic acid (VPA)-, micafungin- and temperature-sensitive phenotypes. (A) The immunosuppressant, chloride, VPA, micafungin and temperature sensitivities of the *its4-1/sip1-i4* mutant cells. Wild-type (wt) and *its4-1/sip1-i4* mutant cells transformed with the multicopy vector pDB248 or the vector containing the *sip1^+^/its4^+^* gene were streaked onto plates containing EMM or EMM plus 0.5 µg/mL FK506, 0.5 µg/mL micafungin, 0.3 M MgCl_2_, or 6 mM valproic acid and then incubated at 27°C for 4 d or at 36°C for 3 d. (B) Schematic representation of the mutation site of the *its4-1/sip1-i4* mutation.

In this study, we identified a novel mutant allele of the *sip1^+^* gene, *sip1-i4*, which was an immunosuppressant- and temperature-sensitive mutant. We demonstrated that Sip1 played an important role in Golgi/endosomal trafficking, but not in endocytosis, and that Sip1 recruited the AP-1 complex to endosomal membranes by physically interacting with this AP-1 complex. Further, we identified Bgs1, required for cell wall synthesis, as the new cargo of AP-1-dependent trafficking.

**Figure 2:**
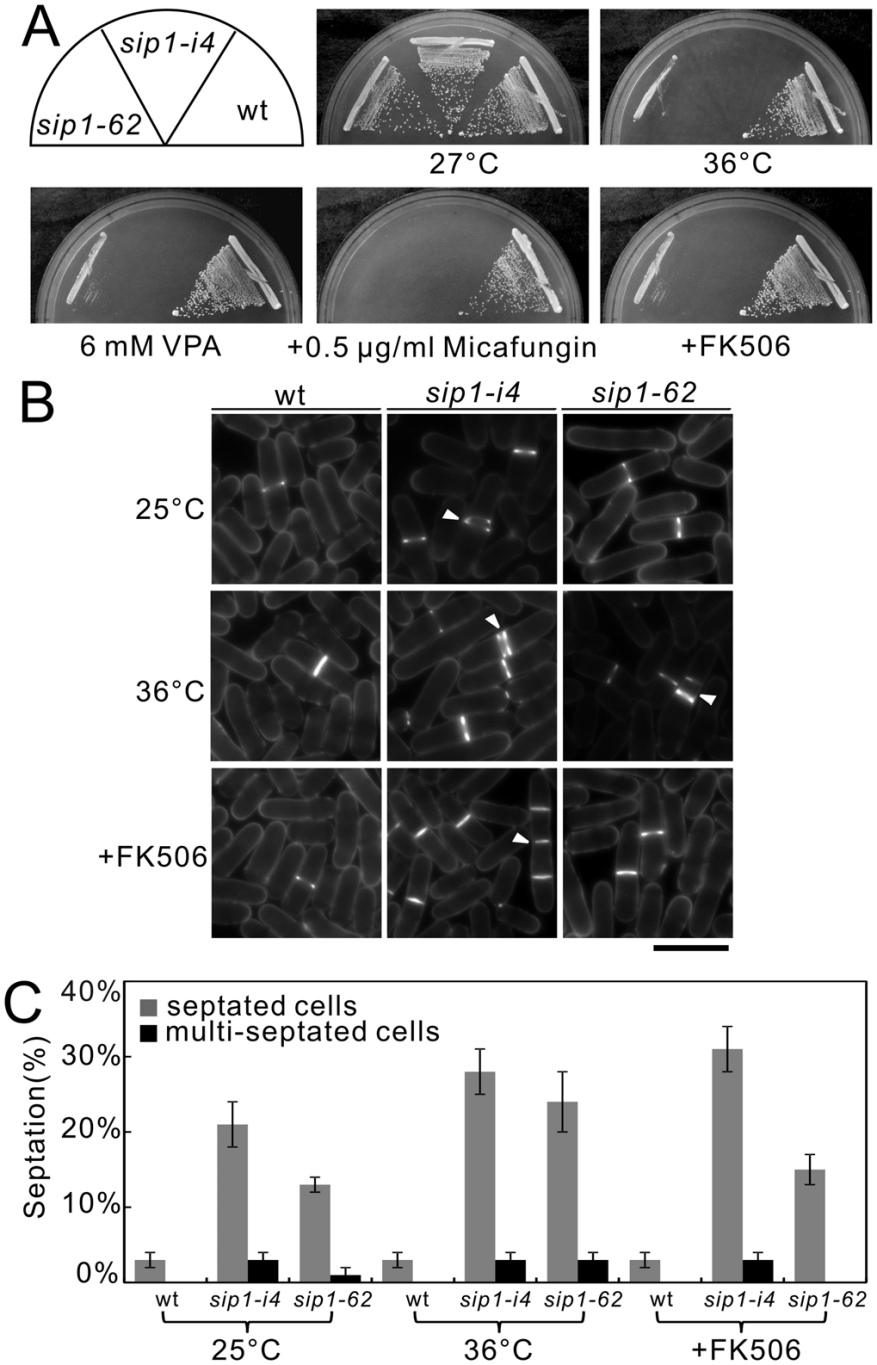
*sip1-62* mutant cells exhibit immunosuppressant-, micafungin-, and temperature-sensitive phenotypes similar to those of *sip1-i4* mutant cells. (A) Immunosuppressant, micafungin, valproic acid and temperature sensitivities of *sip1-62* mutant cells. Wild-type cell (wt), *sip1-i4* mutant cells (*sip1-i4*), and *sip1-62* mutant cells (*sip1-62*) were streaked onto plates containing YES or YES plus 0.5 µg/mL FK506, 0.5 µg/mL micafungin, or 6 mM valproic acid, followed by incubated at 27°C for 4 d or at 36°C for 3 d. (B) The *sip1-i4* mutant cells and *sip1-62* mutant cells are defective in cytokinesis. Wild-type cell (wt), *sip1-i4* mutant cells (*sip1-i4*), and *sip1-62* mutant cells (*sip1-62*) were incubated at 25°C for 1 h, at restrictive temperature (36°C) for 1 h, or FK506 was added for 1 h after the cells were incubated at 25°C for 5 h, and then stained with calcofluor to visualize cell wall and septum. Arrowheads indicated the multi-septated cells. Bar, 10 µm. (C) Septation index and percentage of multi-septated cells in wild-type cell (wt), *sip1-i4* mutant cells (*sip1-i4*), and *sip1-62* mutant cells (*sip1-62*). Cells in C were incubated as in B. The data represent means ± standard deviations of three independent experiments. At least 50 cells were observed at one time in each experiment.

## Materials and Methods

### Strains, Media, Genetic and Molecular Biology Methods

*The Schizosaccharomyces pombe* strains used in this study are listed in Table 1. The complete and minimal media used were yeast extract-peptone-dextrose (YPD) and Edinburgh minimal medium (EMM), respectively [20]. Standard genetic and recombinant DNA methods [21] were used except where stated otherwise. FK506 was provided by Astellas Pharma Inc. (Tokyo, Japan). Genome DNA clones were provided by the National Bio Resource Project, Yeast Genetic Resource Center (Graduate School of Science, Osaka City University).

**Figure 3:**
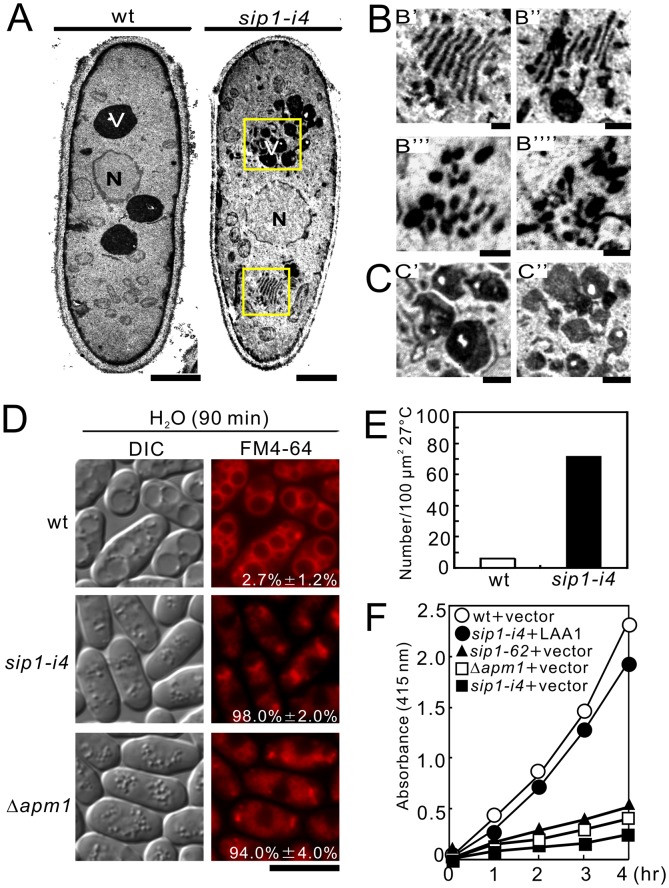
Electron microscopic analysis of *sip1-i4* mutant cells. *sip1-i4* mutant cells exhibited enlarged Golgi structures, accumulated large vesicles, fragmented vacuoles, and defects in acid phosphatase secretion. (A) Wild-type cells (wt) and *sip1-i4* mutant cells (*sip1-i4*) were analyzed by electron microscopy at 27°C. The boxed regions in (wt) were enlarged in B. N, nucleus; V, vacuoles. Bar, 1 µm. (B) Multi-lamellar Golgi structures in *sip1-i4* mutant cells (B' and B''). Putative post-Golgi vesicles around Golgi structures in *sip1-i4* mutant cells (B''' and B''''). Bar, 0.25 µm. (C) Fragmented vacuoles in *sip1-i4* mutant cells (C' and C''). Bar, 0.25 µm. (D) *sip1-i4* mutant cells were defective in vacuole fusion. Wild-type cells (wt), *sip1-i4* mutant (*sip1-i4*), and Apm1-deletion cells (Δ*apm1*) were grown in YPD medium at 27°C. Cells were harvested, labeled with FM4-64 fluorescent dye, resuspended in water, and examined by fluorescence microscopy. Photographs were taken after 90 min. Bar, 10 µm. The number in the image indicates the percent of cells with fragmented vacuoles. Data from at least three independent experiments are expressed as means ± standard deviations. (E) Quantification of large putative post-Golgi vesicles in wild-type and *sip1-i4* mutant cells, Wild-type (wt) and *sip1-i4* mutant cells (*sip1-i4*) were cultured at 27°C for 8 h. Bars represent the mean number of large vesicles in 30-cell sections. Data were normalized to a density per 100 µm^2^. (F) Defective secretion of acid phosphatase in *sip1-i4* mutant cells. Wild-type (wt), *sip1-i4* mutant (*sip1-i4*), *sip1-62* mutant (*sip1-62*), Apm1-deletion (Δ*apm1*), and *sip1-i4* mutant cells (*sip1-i4*), which were transformed with *pRS315-LAA1* plasmids, were assayed for secreted acid phosphatase activity as indicated. The data presented were representative of three independent experiments.

### Isolation of *its4-1/sip1-i4* Mutants

The *its4-1* mutant was isolated during a screen of cells that had been mutagenized with nitrosoguanidine. Strain HM123 cells were mutagenized with 300 µm nitrosoguanidine (Sigma) for 60 min (approximately 10% survival), as described by Moreno *et al.* Mutants were spread on YPD plates to product approximately 1,000 cells/plate and incubated at 27°C for 4 days. The plates were then replica plated at 36°C to plates containing 0.5 µg/ml FK506. Mutants that showed both FK506 sensitivity and temperature sensitivity were selected. The original mutants that were isolated were back-crossed three times to wild-type strains HM123 and HM528.

**Figure 4:**
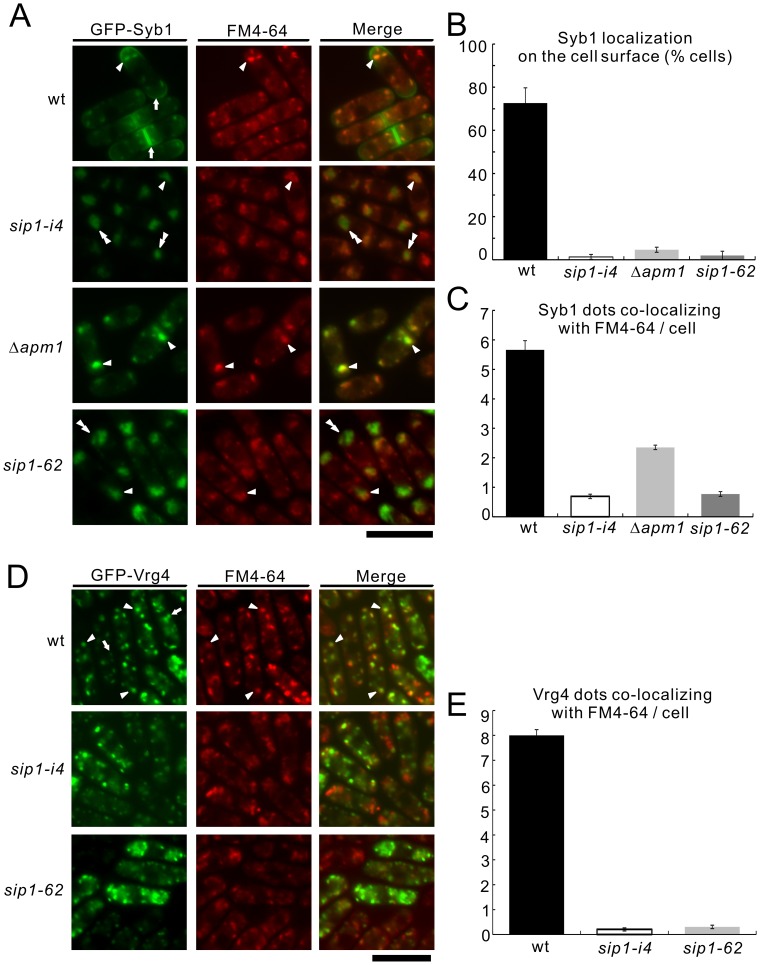
The colocalization of Syb1 and Vrg4 with FM4-64 in wild-type and *sip1-i4* mutant cells. (A) GFP-fused synaptobrevin failed to localize on the cell surface but partially instead accumulated at the Golgi/endosomes in *sip1-i4* mutant cells similar to those seen in Apm1-deletion cells (Δ*apm1*). Wild-type (wt), *sip1-i4* mutant (*sip1-i4*), *sip1-62* mutant (*sip1-62*), and Apm1-deletion cells (Δ*apm1*) expressing GFP-Syb1 were cultured in YPD medium at 27°C were incubated with the dye FM4-64 for 5 min at 27°C to visualize the Golgi/endosomes. The GFP-Syb1 localization and the FM4-64 fluorescence were examined under the fluorescence microscope. Arrowheads indicated the dot-like structures of GFP-Syb1 and Golgi/endosomes stained with FM4-64, double arrowheads indicated the cytoplasmic accumulation, and arrows pointed to the concentrated fluorescence at the medial region and cell surface. Bar, 10 µm. (B) Percentages of cells in which Syb1 was localized at the cell surface, in wild-type cells (wt), *sip1-i4* mutant (*sip1-i4*), *sip1-62* mutant (*sip1-62*), and Apm1-deletion cells (Δ*apm1*). (C) Quantitative analysis of the number of Syb1 dots that co-localized with FM4-64/cell in wild-type (wt), *sip1-i4* mutant (*sip1-i4*), *sip1-62* mutant (*sip1-62*) and Apm1-deletion cells (Δ*apm1*). Cells in B and C were incubated as in A. (D) GFP-fused Vrg4 did not localize to the Golgi/endosomes in *sip1-i4* mutant cells. Wild-type (wt), *sip1-i4* mutant (*sip1-i4*), and *sip1-62* mutant cells (*sip1-62*) expressing GFP-Vrg4 were cultured in YPD medium at 27°C were incubated with the dye FM4-64 for 5 min at 27°C to visualize the Golgi/endosomes. The GFP-Vrg4 localization and the FM4-64 fluorescence were examined under the fluorescence microscope. Arrowheads indicated the dot-like structures of GFP-Vrg4 and Golgi/endosomes stained with FM4-64, and arrows pointed to the dot-like structures of GFP-Vrg4 that did not co-localize with FM4-64. Bar, 10 µm. (E) Quantitative analysis of the number of Vrg4 dots that co-localized with FM4-64/cell in wild-type (wt), *sip1-i4* mutant (*sip1-i4*), and *sip1-62* mutant cells (*sip1-62*). Cells in E were incubated as in D. The data represent means ± standard deviations as the average of three independent experiments with 150 cells in B, C and E.

### Cloning of the *sip1^+^* Gene and Construction of Tagged Its4 Strains

To clone *its4^+^* gene, *its4-1* mutant (SP733) was transformed using an *S*. *pombe* genomic DNA library constructed in the vector pDB248 [22]. Leu+ transformants were replica-plated onto YPD plates at 36°C, and plasmid DNA was recovered from transformants that exhibited plasmid-dependent rescue. Plasmids that complemented the temperature sensitivity of the *its4-1* mutant were cloned and sequenced. Suppressing plasmids contained *sip1^+^* (SPBC27B12.08). The *sip1* ^+^ gene complemented the immunosuppressant sensitivity and sensitivity to various drugs such as FK506, micafungin, and valproic acid as well as all defects in membrane trafficking such as secretion defects and Golgi/endosomal trafficking defects as evidenced by mislocalization of Syb1 and Vrg4, and vacuole fragmentation associated with *its4-1* mutant cells.

**Figure 5:**
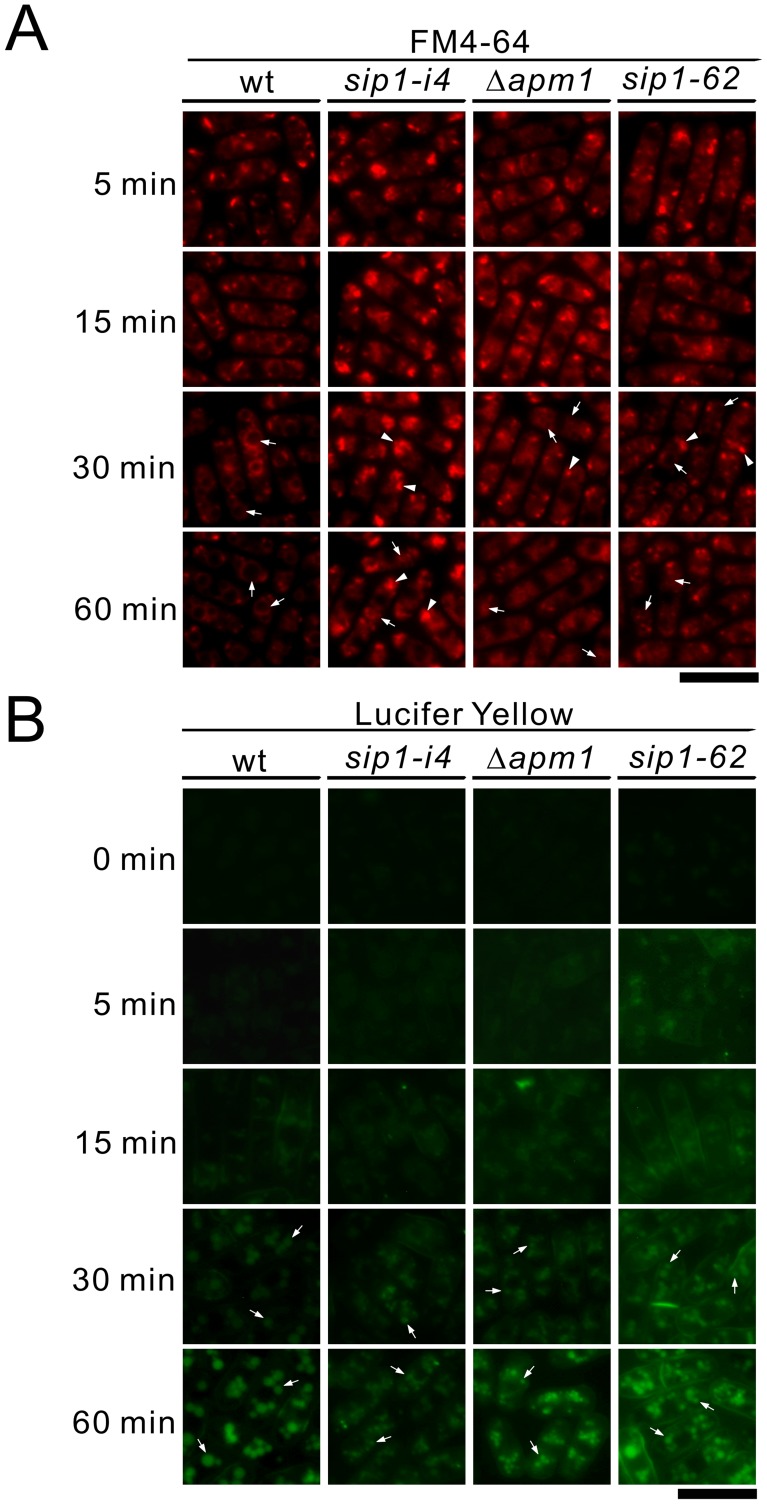
Vacuole fusion was defective, but the internalization step of endocytosis was not impaired in *sip1-62* mutant cells and *sip1-i4* mutant cells. (A) Time-course analysis of FM4-64 internalization. Wild-type (wt), *sip1-i4* mutant (*sip1-i4*), *sip1-62* mutant (*sip1-62*), and Apm1-deletion cells (Δ*apm1*) were incubated in YPD media with FM4-64 at 27°C for 5 min. Washed cells were observed with a fluorescence microscope (FM4-64) at each time point. (B) Time-course analysis of Lucifer yellow (LY) internalization. Wild-type (wt), *sip1-i4* mutant (*sip1-i4*), *sip1-62* mutant (*sip1-62*), and Apm1-deletion cells (Δ*apm1*) were incubated in YPD medium containing Lucifer yellow (5 mg/ml) and were processed as indicated in 5A. Bar, 10 µm.

For the ectopic expression of proteins, we used the thiamine-repressible *nmt1* promoter [23]. Expression was repressed by the addition of 4 µM thiamine to EMM. The carboxy- and amino-terminal epitope-tagged proteins were generated via chromosomal integration of polymerase chain reaction (PCR)-amplified fragments [24]. The C-terminally tagged Its4 strain used in this study behaved like non-tagged parental strains with regard to temperature-sensitivity, immunosuppressant-sensitivity, and sensitivity to drugs including micafungin, indicating that tagging does not interfere with protein function (Figure S1).

### Microscopy and Miscellaneous Methods

Light microscopy methods (e.g., fluorescence microscopy) were performed as described previously [16]. Furthermore, FM4-64 labeling, localization of GFP-Syb1, measurement of acid phosphatase secretion, and conventional electron microscopy were performed as described previously [16].

**Figure 6:**
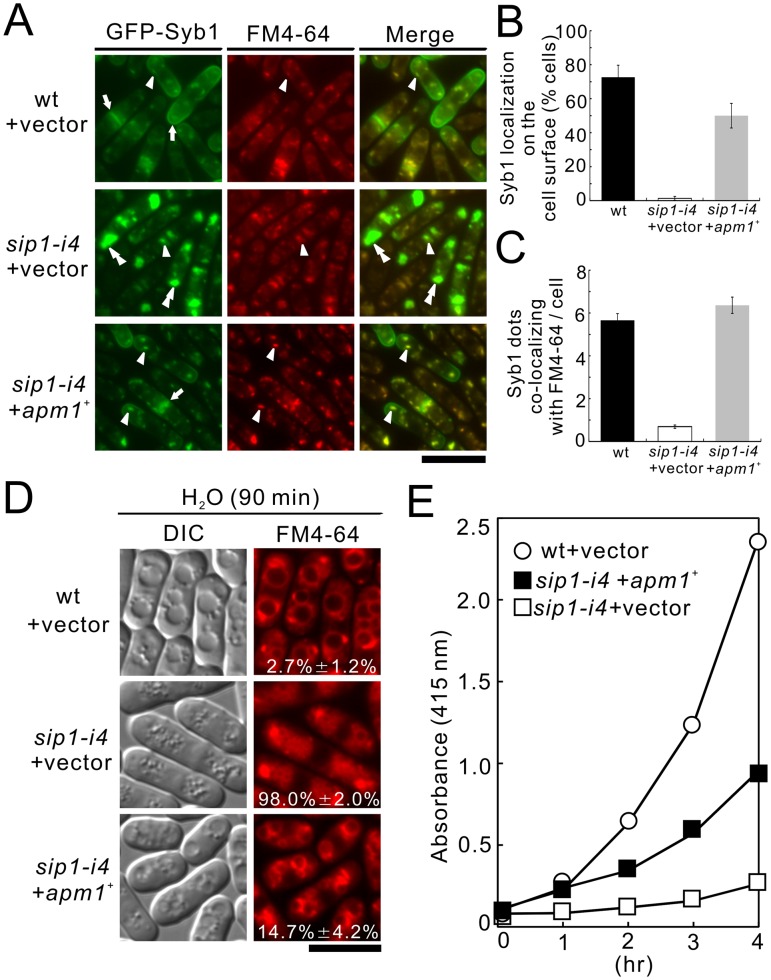
Apm1 suppressed various phenotypes associated with *sip1-i4* mutant cells. (A) Apm1 suppressed the defective localization of GFP-Syb1 in *sip1-i4* mutant cells. Wild-type (wt) and *sip1-i4* mutant cells (*sip1-i4*) expressing GFP-Syb1 cells transformed with pDB248 or the vector containing *apm1* ^+^ was cultured in YPD medium at 27°C. The GFP-Syb1 localization was examined under the fluorescence microscope. Arrowheads indicated the dot-like structures of GFP-Syb1 and Golgi/endosomes stained with FM4-64, double arrowheads indicated the cytoplasmic accumulation, and arrows pointed to the concentrated fluorescence at the medial region and cell surface. Bar, 10 µm. (B) Percentage of cells in which Syb1 was localized at the cell surface. (C) Quantitative analysis of the number of Syb1 dots that co-localized with FM4-64/cell. Cells in B and C were the same as indicated in A. (D) Apm1 suppressed the defective in vacuole fusion in *sip1-i4* mutant cells. Wild-type (wt) and *sip1-i4* mutant cells (*sip1-i4*) transformed with pDB248 or the vector containing *apm1* ^+^ cultured in YPD medium at 27°C. Cells were collected, labeled with FM4-64 fluorescent dye for 60 min, resuspended in water, and examined by fluorescence microscopy. Bar, 10 µm. The number in the image indicates the percentage of cells with fragmented vacuoles. Data from at least three independent experiments are expressed as means ± standard deviations. (E) Apm1 suppressed the defective secretion of acid phosphatase in *sip1-i4* mutant cells. Wild-type (wt) and *sip1-i4* mutant cells (*sip1-i4*), which were transformed with either the pDB248 vector or the *apm1* ^+^-containing vector, were assayed for acid phosphatase activity. The data presented were representative of three independent experiments.

### Image Quantification

All the image quantifications were done for 3 individual datasets which summed up to 150 counted cells.

**Figure 7:**
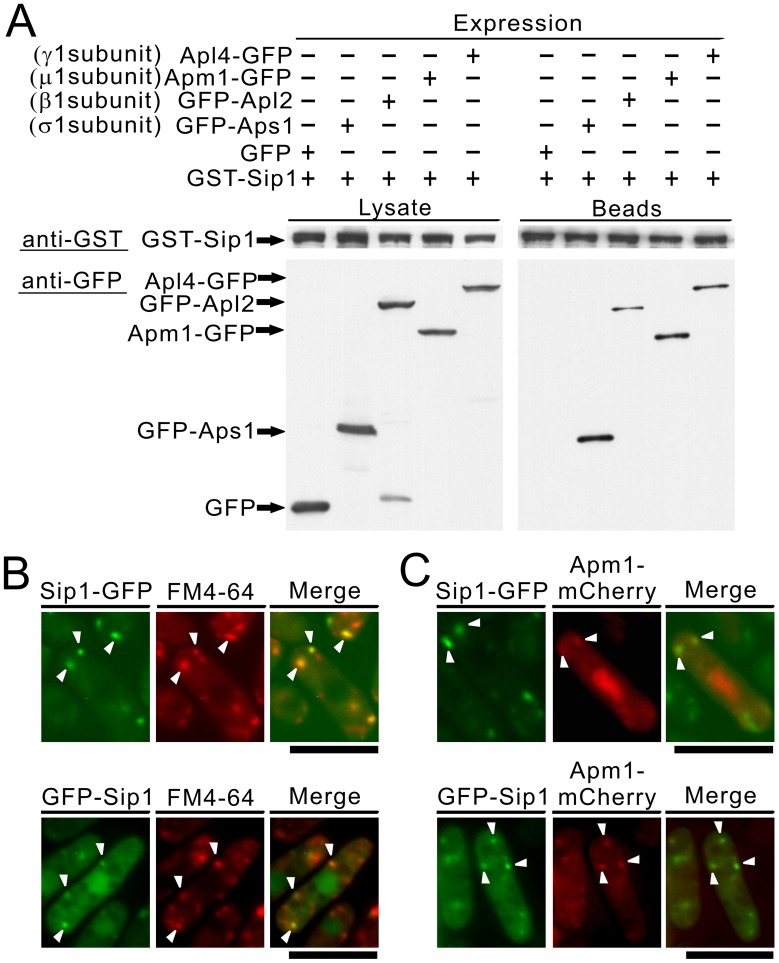
Sip1-GFP localized at the Golgi/endosome and Sip1 associated with the 4 subunits of the AP-1 complex. (A) Binding assay involving Sip1 and the 4 subunits of the AP-1 complex. GST pull-down experiments performed with expressing chromosome-borne GST-Sip1 under the control of the *nmt1* promoter; cells expressing GFP alone or Apm1-GFP, GFP-Apl2, Apl4-GFP or GFP-Aps1 were collected, and the lysates were incubated with purified full-length Sip1 fused GST protein. GST-tagged Sip1 was precipitated by glutathione beads, washed extensively, subjected to SDS-PAGE, and immunoblotted using anti-GFP or anti-GST antibodies. (B) The colocalization of Sip1-GFP or GFP-Sip1 with FM4-64 in wild-type cells. Wild-type (wt) cells, expressing chromosome-borne Sip1-GFP or expressing chromosome-borne GFP-Sip1 under the control of the *nmt1* promoter, were examined by fluorescence microscopy under the repressed conditions. The cells were incubated with FM4-64 fluorescent dye for 5 min at 27°C to visualize the Golgi/endosomes. The fluorescence of the FM4-64 was examined under the fluorescence microscope. Arrowheads indicated the dot-like structures and Golgi/endosomes. Bar, 10 µm. (C) The partial colocalization of Sip1-GFP or GFP-Sip1 with Apm1-mCherry in wild-type cells. Wild-type cells expressing chromosome-borne Sip1-GFP or expressing chromosome-borne GFP-Sip1 under the control of the *nmt1* promoter, were transformed with pREP1-Apm1-mCherry. The cells were cultured in EMM medium at 27°C and examined by fluorescence microscopy. Arrowheads indicated the colocalization of Sip1-GFP or GFP-Sip1 with Apm1-mCherry at Golgi/endosomes. Bar, 10 µm.

**Figure 8:**
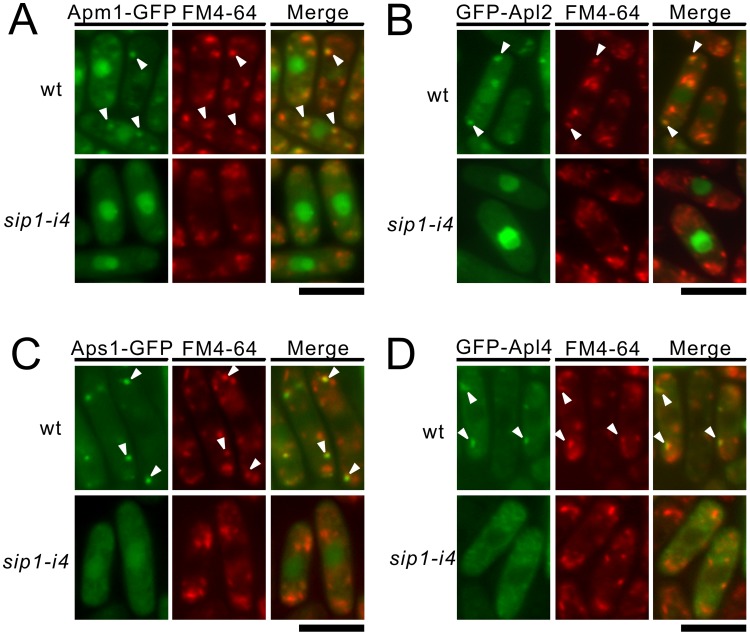
The individual adaptin subunit failed to localize at Golgi/endosomes in *sip1-i4* mutant cells. Subcellular localization of Apm1-GFP (A), Aps1-GFP (C), GFP-Apl2 (B), and GFP-Apl4 (D) in wild-type (wt) and *sip1-i4* mutant cells (*sip1-i4*). Cells expressing various GFP-tagged adaptin subunits were cultured in YPD medium at 27°C were incubated with the dye FM4-64 for 5 min at 27°C to visualize the Golgi/endosomes. The fluorescence of the FM4-64 was examined under the fluorescence microscope. Arrowheads indicated the localization of GFP-tagged adaptin subunits to the Golgi/endosomes. Bar, 10 µm.

### Staining of Vacuoles with Lucifer Yellow

The staining with Lucifer yellow is described in [16]. Briefly, cells were grown to an exponential phase in YES medium, harvested with centrifugation for 3 min at 4°C, resuspended in fresh YES medium containing 5 mg/ml Lucifer yellow carbonyl hydrazine (Sigma-Aldrich), and incubated at 27°C for various periods in time-course experiments. Aliquots were harvested at times indicated, washed three times with the medium, and fluid-phase endocytosis was microscopically observed under the fluorescence microscope.

**Figure 9:**
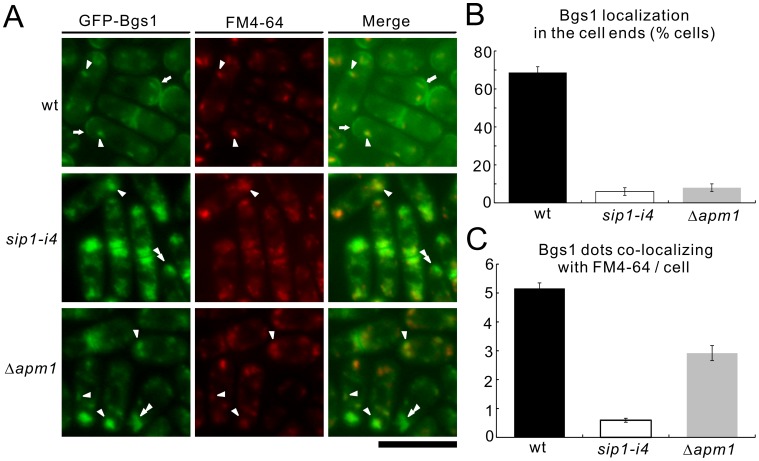
Colocalization of Bgs1 with FM4-64 in wild-type, *sip1-i4* mutant, and Δ*apm1* cells. (A) Bgs1 did not localize at cell ends, although it partially accumulated in the Golgi/endosomes in *sip1-i4* mutant and Δ*apm1* cells. Wild-type (wt), *sip1-i4* mutant (*sip1-i4*), and Apm1-deletion cells (Δ*apm1*), which expressed chromosome-borne GFP-Bgs1, were examined by fluorescence microscopy under the repressed conditions. Cells were cultured in EMM at 27°C for 6 h. GFP-Bgs1 localization and FM4-64 fluorescence were examined under a fluorescence microscope. Arrowheads indicate the dot-like structures of GFP-Bgs1 and Golgi/endosomes stained with FM4-64; double arrowheads indicated cytoplasmic accumulation, and arrows highlight the concentrated fluorescence at the medial region and cell ends. Bar, 10 µm. (B) Percentage of cells in which Bgs1 was localized at cell ends in wild-type (wt), *sip1-i4* mutant (*sip1-i4*), and Apm1-deletion cells (Δ*apm1*). (C) Quantitative analysis of the number of Bgs1 dots co-localizing with FM4-64/cell in wild-type (wt), *sip1-i4* mutant (*sip1-i4*), and Apm1-deletion cells (Δ*apm1*). Cells in B and C were incubated as in A. The data represent means ± standard deviations as the average of three independent experiments with 150 cells in B and C.

## Results

### Isolation of the *its4-1/sip1-i4* Mutant

To isolate new molecules that function in membrane trafficking, we searched for mutants sensitive to the immunosuppressive drug FK506 and isolated the *its4-1* mutant. As shown in Figure 1A, *its4-1* mutants grew equally well compared with the wild-type cells at the permissive temperature of 27°C. However, *its4-1* mutant cells failed to grow at either the restrictive temperature of 36°C or, in media containing either 0.3 M MgCl_2_ or FK506, a specific inhibitor of calcineurin phosphatase, whereas the wild-type cells grew well (Figure 1A, *sip1-i4*+vector). The *its4-1* mutants also showed greater sensitivity to the cell wall-damaging agent micafungin and to valproic acid than did wild-type cells (Figure 1A, *sip1-i4*+vector).

### The *its4-1* is an Allele of the *sip1^+^* Gene Encoding Homolog of the Clathrin-associated Adaptor Protein-1 (AP-1) Complex-Interacting Protein

The *its4* ^+^ gene was cloned by complementation of the temperature-sensitive growth defect of *its4-1* mutant cells (Figure 1A, *sip1-i4*+*its4* ^+^). *its4* ^+^ also complemented sensitivities to drugs associated with the *its4-1* mutant (Figure 1A).

Nucleotide sequencing of the cloned DNA fragment revealed that the *its4* ^+^ gene was identical to the *sip1* ^+^ gene (SPBC27B12.08), that encodes a 1919 amino acid protein, which shares amino acid similarities with *Homo sapien* HEATR5B (309/1919, 16.1% identity) and *S. cerevisiae* Laa1 (329/1919, 17.1% identity). Linkage analysis was performed (see Materials and Methods); it indicated allelism between the *sip1* ^+^ gene and the *its4-1* mutation. We therefore renamed *its4-1* as *sip1-i4*.

To characterize the mutation site in the *sip1-i4* allele, genomic DNA from the *sip1-i4* mutant was isolated, and the full-length coding region of the *sip1-i4* was sequenced. The G-to-A nucleotide substitution caused a serine to be altered to a termination codon at the amino acid position 1434, resulting in a truncated protein product lacking 485 amino acids downstream of the mutation (Figure 1B).

As Jourdain *et al*. identified the *sip1-62* mutant, an independent allele of the *sip1^+^* gene [19], it is intriguing to investigate the phenotypic differences between the two *sip1* mutant alleles. As shown in Figure 2A, both *sip1-62* and *sip1-i4* mutant cells exhibited sensitivities to temperature, valproic acid, micafungin, and the immunosuppressive drug FK506, although the *sip1-i4* mutant cells exhibited more pronounced sensitivity to the stresses examined (Figure 2A).

In addition, we examined the effect of the *sip1-i4* mutation on cytokinesis, as *sip1-62* mutants were reported to show defects in cytokinesis [19]. For this, we monitored the septation index of the two *sip1* mutants at the permissive and the restrictive temperatures. The *sip1-i4* mutant cells showed a significantly higher septation index than the wild-type and the *sip1-62* mutant cells, indicating that both *sip1* mutant cells had defects in cytokinesis (Figure 2B, 2C). Notably, FK506 treatment for 1 hr induced the appearance of multi-septated phenotypes in *sip1-i4* cells, but not in *sip1-62* cells (Figure 2B, arrowheads, Figure 2C). Thus, the effect of FK506 treatment on cytokinesis was more pronounced in *sip1-i4* cells than in *sip1-62* cells (Figure 2B, 2C). This may be consistent with the finding that *sip1-i4* cells exhibited more pronounced sensitivity to FK506 than did *sip1-62* cells at 27°C.

### Electron Microscopic Analysis of *sip1-i4* Cells

Sip1 was previously identified as a novel F-box protein interactor essential for cytokinesis and endocytosis [19]. In order to gain a detailed insight into the role of Sip1 in membrane trafficking, we analyzed *sip1-i4* cells with electron microscopy. In general, electron microscopic analysis of mutants that exhibit defects in membrane trafficking reveals the accumulation of organelles or vesicular intermediates of the compartments that precede the step in which they first function [25], [26], [27]. Wild-type and *sip1-i4* cells were cultured at 27°C and examined by electron microscopy in order to determine whether *sip1-i4* cells accumulate such organelles or vesicular structures (Figure 3A and 3B). Notably, Golgi structures in *sip1-i4* cells were thick, swollen, and frequently multi-lamellar (Figure 3B, B’ and B’’). Moreover, large vesicular structures (diameter, >100 nm) associated with Golgi stacks were observed in *sip1-i4* cells (Figure 3B, B’’), suggesting that Sip1 is involved in vesicle formation at the trans-Golgi network. These structures were rarely found in wild-type cells (Figure 3A, wt). Moreover, *sip1-i4* cells accumulated putative large post-Golgi vesicles ranging from 100 to 150 nm in size that were intensely stained after permanganate fixation (Figure 3B, B’’’, B’’’’, and 3E); on the other hand, the number of vesicles observed in wild-type cells was extremely low (Figure 3A and 3E). Another striking feature of *sip1-i4* cells is that their vacuoles were fragmented (Figure 3A, and 3C, C’, C’’) as compared with those of wild-type cells (Figure 3A, wt). Consistently, *sip1-i4* cells exhibited vacuolar fusion defects induced by osmotic stress, in a similar fashion to Δ*apm1* cells. Hypotonic stress causes a dramatic fusion of vacuoles in wild-type, but not in *sip1-i4* or Δ*apm1* cells (Figure 3D), indicating that Sip1 is required for vacuole fusion. Quantification of the percentages of cells with fragmented vacuoles indicated that more than 90% of the *sip1-i4* (98.0% ±2.0%) and Δ*apm1* (94.0% ±4.0%) cells had fragmented vacuoles, whereas less than 5% of the wild-type cells (2.7% ±1.2%) had fragmented vacuoles (Figure 3D).

Consistent with the accumulation of putative large post-Golgi vesicles, *sip1-i4* cells, similar to Δ*apm1* cells, showed defects in acid phosphatase secretion (Figure 3F). In addition, LAA1 overexpression suppressed these secretion defects in *sip1-i4* cells, suggesting that Sip1 shared a functional homology with Laa1 (Figure 3F, *sip1-i4*+LAA1). It should be noted that abnormal findings seen in the electron micrographs of *sip1-i4* cells are similar to the previously reported findings in the *ypt3-i5* mutant [27] and Δ*apm1* cells [16]. This suggested that the role of Sip1 in membrane trafficking was associated with Golgi/endosomes and vacuoles.

### *sip1* Mutant Cells Exhibit Defects in Golgi/Endosomal Trafficking

The aforementioned results strongly suggested that defects in the membrane trafficking in *sip1-i4* and Δ*apm1* cells were closely related. Thus, we examined the phenotypes of *sip1-i4* cells in greater detail and compared these phenotypes with those of the *sip1-62* cells identified by Jourdain *et al* [19]. First, we visualized Syb1 as a GFP-fusion protein in these cells. Syb1, the synaptobrevin in fission yeast, is a vesicle-associated membrane protein that can be copurified with secretory vesicles [28]. In order to assess the Golgi-to-endosome or Golgi-to-plasma membrane trafficking pathway, the localization of GFP-Syb1 was monitored.

As shown in Figure 4A, GFP-Syb1 fluorescence observed in the cytoplasm of wild-type cells was detected as a small punctuate pattern and was co-localized with FM4-64 dots at an early stage of endocytosis (arrowhead, wt); this fluorescence pattern was also enriched in the cell ends and the medial region (arrows, wt). In contrast, GFP-Syb1 did not localize at the cell surface and the cell ends of *sip1-i4*, *sip1-62* and Δ*apm1* cells (Figure 4A, 4B). Instead, GFP-Syb1 fluorescence was observed as large dot-like structures that co-localized with FM4-64 in Δ*apm1* cells (Figure 4A, arrowheads, 4C) and partly co-localized with FM4-64 in *sip1-i4* and *sip1-62* cells (Figure 4A, double arrowheads, 4C). This suggested that, as with Apm1, Sip1 was involved in the Golgi/endosome membrane trafficking pathway. Quantification of Syb1 localization in the cell ends and Syb1 co-localization with FM4-64 confirmed these results (Figure 4B, 4C).

To further characterize the defects in membrane trafficking associated with the Golgi/endosome system in *sip1* mutant cells, we investigated the co-localization of the Golgi marker protein Vrg4 [29] and FM4-64. In wild-type cells, GFP-Vrg4 mostly co-localized with FM4-64 (Figure 4D, wt, arrowheads). However, a small number of the Vrg4 positive dots did not co-localize with FM4-64 (Figure 4D, wt, arrow). In contrast, in *sip1-i4* and *sip1-62* cells, the punctuate fluorescence pattern of Vrg4 did not co-localize with FM4-64 (Figure 4D). Quantification of Vrg4 co-localization with FM4-64 showed that 8.0±0.2 of the Vrg4 punctates co-localized with FM4-64 fluorescence in wild-type cells, whereas 0.2±0.1, and 0.2±0.1 of the Vrg4 punctates co-localized with FM4-64 in *sip1-i4* and *sip1-62* cells, respectively (Figure 4E).

In budding yeast, the GDP mannose transporter predominantly localizes to the Golgi apparatus as detected by indirect immunofluorescent staining [30]. Abe *et al*. also investigated the mechanism of Golgi-resident Vrg4 protein in the stream of vesicular traffic, and found that Vrg4p is recycling between the ER and Golgi apparatus [31]. In S. *pombe*, our previous paper showed that Vrg4 mostly co-localized with the Golgi marker Krp1, which suggested that Vrg4 was predominantly localized to the Golgi, similar to what is observed in budding yeast [17]. Thus, we hypothesize that the Vrg4-positive dots that co-localize with FM4-64 may represent Golgi, and that the Vrg4-positive dots that did not co-localize with FM4-64 may represent unidentified structures, including the ER. Altogether, these results indicated that both *sip1* mutant alleles resulted in defects in Golgi/endosomal membrane trafficking.

### Sip1 is not Essential for Endocytosis

To determine if the endocytosis defects reported by Jourdain *et al*. were specific to the *sip1-62* mutant allele, we performed a time-course experiment using FM4-64, a vital dye that is internalized in living cells through endocytosis and accumulates in vacuoles [32]. As a control, we used Δ*apm1* cells in which endocytosis was not impaired [16]. In wild-type cells, small fluorescent dots appeared in the cytoplasm within 5 min of incubation at 27°C (Figure 5A, 5 min, wt); these dots became increasingly brighter over the next 10 min (Figure 5A, 15 min, wt). The staining of these endosomal intermediates subsequently decreased concomitant with the appearance of FM4-64 in vacuolar membranes (Figure 5A, 30 min, wt, arrows). After 60 min, FM4-64 staining was predominant in the vacuoles (Figure 5A, 60 min, wt, arrows).

In *sip1-i4*, *sip1-62* and Δ*apm1* cells, FM4-64-labeled endosomal intermediates had kinetics similar to that observed in wild-type cells, indicating that the internalization step of endocytosis was not impaired in these mutants (Figure 5A, 5 min, 15 min, 30 min). Subsequent delivery to the vacuole was not impaired in these mutant cells, although some FM4-64 fluorescent dots were observed in most of the *sip1-i4* mutant (98.01% ±2.01%) and the *sip1-62* mutant cells (85.31% ±4.21%). By comparison, the appearance of these dots was less frequent in wild-type cells (16.01% ±2.01%) and Δ*apm1* cells (38.71% ±3.1%). This suggested that there were defects in post-endocytic endosomal trafficking toward the vacuole in *sip1* mutants (Figure 5A, 60 min, arrowheads). Because the vacuoles in *sip1* and Δ*apm1* mutants were smaller and highly fragmented (Figure 5A, 60 min, arrows), it could not be definitely concluded that the dye had reached up to the vacuoles or the late endosomes.

Therefore, we performed another time-course experiment using the membrane-impermeable fluorescence dye Lucifer yellow to monitor the fluid phase of endocytosis [33]. After 30 min incubation, the vacuoles in wild-type, *sip1-i4*, *sip1-62* and Δ*apm1* cells were observed as fluorescence-positive compartments (Figure 5B, 30 min, arrows), and there was no significant delay in dye uptake over the time course in these mutant cells. In contrast, Lucifer yellow uptake was not observed in the endocytosis-defective mutant, as was the case for a *ypt7* disruption strain [34] (our unpublished data). Therefore, we concluded that Sip1 was not essential for endocytosis but it was involved in post-endocytic endosomal trafficking towards the vacuole.

### Sip1 Shows Genetic Interactions with Apm1

To investigate the functional relationship between Sip1 and Apm1, we investigated the effect of Apm1 overexpression on the membrane-trafficking defects in *sip1-i4* cells. We first examined the effect of Apm1 overexpression on abnormal localization of GFP-fused Syb1 in *sip1-i4* cells. GFP-Syb1 failed to localize on the cell surface (Figure 6A, *sip1-i4*+ vector, arrows, 6B, 1.3% ±1.2%), or Golgi/endosomes in *sip1-i4* cells; instead, they were observed as large, brightly fluorescent dots in the cytoplasm at 27°C (Figure 6A, *sip1-i4*+vector, double arrowheads, 6C). Notably, GFP-Syb1 was visible at the cell ends in *sip1-i4* cells that harbored *apm1* ^+^ (Figure 6A, *sip1-i4+ apm1* ^+^, arrows, 6B, 50.0% ±7.2%), and Apm1 overexpression recovered normal Syb1 dots that co-localized with FM4-64 (Figure 6A, *sip1-i4+ apm1* ^+^, arrowheads, 6C, 6.4±0.4).

Next, we examined the effect of Apm1 overexpression on vacuole fusion observed in *sip1-i4* cells (Figure 3C). After the cells were labeled with FM4-64 for 60 min, collected, washed, and resuspended in water for 90 min, the wild-type cells had large prominent vacuoles resulting from vacuole fusion (Figure 6D, wt + vector, 2.7% ±1.2%). On the other hand, vacuoles in the *sip1-i4* cells remained small and numerous (Figure 6D, *sip1-i4*+ vector, 98.0% ±7.2%), indicating a defect in vacuole fusion. In contrast, the *sip1-i4* cells transformed with *apm1^+^* contained larger vacuoles compared with those harboring the vector alone, indicating that Apm1 overexpression partially, but clearly, suppressed the defects in vacuole fusion observed in *sip1-i4* cells (Figure 6D, *sip1-i4*+ *apm1* ^+^, 14.7% ±4.2%). We further examined the effect of *apm1^+^* overexpression on the secretion defects in associated with *sip1-i4* cells. Overexpression of *apm1^+^* partially, but significantly, stimulated secretion in *sip1-i4* cells (Figure 6E, *sip1*-*i4*+ *apm1* ^+^).

### Sip1 Associates with the AP-1 Complex in *S. pombe*

Next, we examined whether Sip1 could bind to each subunit of the AP-1 complex. To this end, we fused Sip1 to glutathione-*S*-transferase (GST) and overexpressed the fusion protein using an inducible *nmt1* promoter; the induced cells were used to prepare lysates. These lysates were then used in binding experiments in which purified full-length Apm1, Apl2, Apl4, and Aps1 were fused to GFP or to the control GFP protein. Results showed that the Sip1 protein bound to each of the subunits of the AP-1 complex (Figure 7A). Thus, Sip1, like Laa1 in budding yeast [18] and the mammalian homologue p200 [9], acts as an AP-1 complex-interacting protein.

### Sip1 is an Endosomal Protein

Next, we examined localization of Sip1. Experiments were performed with Sip1-tagged with GFP expressed chromosomally under its own promoter, and GFP-tagging at its C-terminus did not affect Sip1-GFP function, which was indicated by the findings that C-terminally tagged Sip1-GFP strain showed no sensitivity to high temperature, or various drugs, including FK506 and micafungin (Figure S1A). Sip1-GFP was localized in the dot-like structures observed in the cytoplasm (Figure 7B, arrowheads), which co-localized with FM4-64 during an early stage of endocytosis. After 5 min of dye uptake, most Sip1-GFP dot-like structures were co-localized with FM4-64-positive structures (Figure 7B, Sip1-GFP, arrowheads). In addition, we constructed a strain in which Sip1 was chromosomally tagged with GFP at its N-terminus and expressed under the *nmt1* promoter. In this strain, as reported by Jourdain *et al*., Sip1 localizes in the nucleus in addition to the dots, which co-localized with FM4-64 during an early stage of endocytosis (Figure 7B, GFP-Sip1, arrowheads) [19]. It should be noted that the differences in the localization of the Sip1 protein in the nucleus plus the Golgi/endosomes may be attributable to the utilization of an inducible *nmt1* promoter and/or differences in tagging of the Sip1 protein wherein GFP was fused to its N-terminus or C-terminus. To allow co-localization with Sip1-GFP, we tagged Apm1 with the fluorescent epitope mCherry and the resultant Apm1-mCherry protein expressed from its endogenous promoter was detectable at the dot-like structures and the nucleus, similar to the findings reported with endogenous Apm1 protein tagged with GFP [16]. When Sip1-GFP and Apm1-mCherry were co-expressed in wild-type cells from their own promoters, some of the punctuate structures of Sip1 co-localized with Apm1 dots in the cytoplasm (Figure 7C; upper panel, arrowheads). Furthermore, we confirmed the co-localization between Sip1 and Apm1 dots in the cytoplasm using *nmt1*-GFP-Sip1 protein (Figure 7C; lower panel, arrowheads). We also evaluated the co-localization of Sip1 with mCherry-tagged Anp1 and Sec72, which are *cis*-Golgi and *trans*-Golgi marker proteins, respectively. Sip1-GFP or GFP-Sip1 did not co-localize with Anp1-mCherry (Figure S1B), and both proteins partially co-localized with Sec72-mCherry (Figure S1C). Together, these results indicated that Sip1 was an endosomal protein, but not an endocytic vesicle protein.

### The AP-1 Complex is Mislocalized in *sip1-i4* Mutant Cell

We next examined the effect of *sip1-i4* mutation on the intracellular localization of the individual subunit of the AP-1 complex, namely Aps1, Apl2, Apm1 and Apl4. As shown in Figure 8, the dot-like fluorescence of Apm1-GFP, GFP-Apl2, Aps1-GFP and GFP-Apl4 co-localized with FM4-64-positive structures in wild-type cells, suggesting that the four adaptin subunits of the AP-1 complex are localized to the Golgi/endosomes. In contrast, in *sip1-i4* mutant cells, Apm1, Apl2, and Aps1 failed to localize to the Golgi/endosomes (Figure 8), as the specific dot-like structures were hardly observed in *sip1-i4* mutant cells. Instead, they localized diffusely in the cytoplasm and were enriched in the nucleus. GFP-Apl4 was observed as numerous punctuate structures in *sip1-i4* mutant cells, but not in the nucleus, although these dot-like structures rarely co-localized with FM4-64; this was in contrast to the situation in the wild-type cells in which most Apl4 dots merged with FM4-64 (Figure 8D, arrowheads). These results suggest that the *sip1-i4* mutation affected the localization of AP-1 complex to the Golgi/endosomes. In a reciprocal experiment, Sip1-GFP was examined in wild-type and the deletion strain of each of the adaptin subunits. Sip1-GFP distribution seemed to be unaltered in either of these deletion mutant cells wherein dot-like structures of Sip1 co-localized with FM4-64. We then tried to determine the localization of Sip1-GFP in Δ*apm1* cells by examining the co-localization of Sip1-GFP with the *cis*-Golgi marker Anp1, and the *trans*-Golgi marker Sec72 and compared these localizations with those in wild-type cells. These results showed that Sip1-GFP partly co-localized with Sec72, but not with Anp1 in Δ*apm1* cells, which suggested that Sip1-GFP was partly localized in the *trans*-Golgi apparatus and partly localized in endosomes in Δ*apm1* cells. These localization patterns were similar to those obtained with the wild-type cells indicating that the AP-1 complex was not required for the localization of Sip1 to Golgi/endosomes (Figure S2).

### Sip1/AP-1 is Required for Transporting Glucan Synthase Bgs1 from Golgi/Endosomes to the Plasma Membrane

What is the mechanism of the FK506 sensitivity observed in *sip1-i4* cells? One possibility is that calcineurin regulates the enzymes involved in cell wall synthesis and/or their breakdown, thereby affecting cell wall integrity. If Sip1/AP-1 is involved in the proper transport/function of cell wall biosynthesis enzymes, the combined defects in the Sip1/AP-1 system and calcineurin may result in lethality. One attractive candidate cargo of AP-1 dependent trafficking is Bgs1/Cps1, because Bgs1 is an enzyme involved in beta 1, 3-glucan synthesis and, therefore, is important for cell wall integrity [35], [36]. In addition, our previous screen identified *bgs1-i2*, a mutant allele of the *bgs1* ^+^ gene, which exhibited temperature- and immunosuppressant sensitivity [37]. Thus, we examined the effects of the loss of Sip1/AP-1 system function on the localization of Bgs1, by visualizing GFP-Bgs1 expressed chromosomally under its own promoter.

In wild-type cells, Bgs1 was localized to the growing ends at 27°C (Figure 9A, wt, arrows), consistent with a previous report [38]. Notably, the dot-like fluorescence pattern of GFP-Bgs1 was also observed in the cytoplasm of wild-type cells. These Bgs1 dots co-localized with FM4-64 in the early stage of endocytosis (Figure 9A, wt, arrowheads), which indicated that Bgs1 was localized to Golgi/endosomes. In contrast, in *sip1-i4* cells, the localization of Bgs1 to the cell ends was impaired, which was also supported by the quantification of Bgs1 localization to the cell ends (Figure 9A, 9B, *sip1-i4*). In addition, Bgs1 was observed as a large cluster in the cytoplasm (Figure 9A, *sip1-i4*, arrowheads) and the number of Bgs1 dots that co-localized with FM4-64 was significantly lower in *sip1-i4* cells (0.6±0.1) as compared with the wild-type cells (5.2±0.2; Figure 9C). In Apm1 deletion cells, the localization of Bgs1 was similar to that observed in *sip1-i4* cells, although the extent of co-localization of Bgs1 dots with FM4-64 was relatively higher compared with *sip1-i4* cells (Figure 9A, 9C, Δ*apm1*). These results suggested that Sip1/AP-1 played a role in the correct sorting of Bgs1 and that Bgs1 was a new cargo of AP-1 dependent trafficking required for correct cell wall synthesis.

## Discussion

In this study, we present several lines of evidence that suggest a role for Sip1 in Golgi/endosomal trafficking as an AP-1 accessory protein. Our study identified β-glucan synthase as a new cargo of AP-1-dependent trafficking required for correct cell wall synthesis.

The *sip1*-*i4* mutants displayed phenotypes resembling those associated with Apm1 deletion, including defects in Golgi/endosomal trafficking and secretion, temperature- and immunosuppressant-sensitive growth, and defects in cell integrity. In addition, the *sip1-i4* mutation affected AP-1 complex localization at Golgi/endosomes, which suggested a conserved role for Sip1 in AP-1 localization. Together with the physical interaction between Sip1 and the AP-1 complex and the co-localization of these proteins *in vivo*, these results strongly suggest that Sip1 is an AP-1 accessory protein. Apm1 overexpression suppressed the *sip1-i4* mutant phenotypes with regard to membrane trafficking, including abnormal Syb1 localization and defects in vacuole fusion and secretion. Although most of the AP-1 complex was mislocalized in the *sip1-i4* mutant cells, overproduction of Apm1 may have increased the amount of the residual AP-1 complex that was localized in the proper organelle, thereby restoring its function. Thus, *sip1-i4* mutant phenotypes can be partly attributed to the loss of proper AP-1 complex function due to its mislocalization.

Importantly, our study demonstrated that Sip1 was not essential for endocytosis and that it was an endosomal and not an endocytic protein. A recent study by Jourdain *et al*. reported that Sip1 was an endocytic vesicle protein that was important for endocytosis [19]. Jourdain *et al.* assigned Sip1 to endocytic vesicle, since it did not co-localize with a Golgi marker (Anp1), it was only partially localized with a *trans*-Golgi marker (Sec72), and it co-localized with internalized FM4-64. This pattern could also describe endosomal localization. Our study demonstrated Sip1 endosomal localization based on its co-localization with Apm1 (endosome) and FM4-64 at an early stage of endocytosis.

The *sip1-i4* mutation resulted in a termination codon at amino acid position 1434 located within the highly conserved region (HCR), which contains an approximately 200-amino acid segment conserved throughout evolution in this protein family [18]. This mutation resulted in a truncated protein product that lacked 485 amino acids at the C-terminus of the Sip1 protein (corresponding to the last 41 amino acids of the HCR). Studies of budding yeast Laa1 showed that the HCR was dispensable for its interaction with AP-1, but was necessary for the localization of Laa1 to the punctuate structures [18]. Therefore, the Sip1-i4 mutant protein may behave like Laa1ΔHCRp and, thus, supports the importance of HCR in AP-1 localization. This also narrows down the region within the HCR that is important for AP-1 localization. In addition, the effect of the *sip1-i4* mutation was more pronounced than the budding yeast Laa1ΔHCRp, as the AP-1 complex appeared to be almost completely mislocalized in cells with the *sip1-i4* mutation, even during the early logarithmic phase.

We also showed that both the *sip1-i4* and *sip1-62* mutants displayed similar phenotypes, including hypersensitivity to temperature, the immunosuppressive drug FK506, valproic acid, and micafungin (Figure 2). In addition, both mutant cells exhibited cytokinesis defect as evidenced by an increased septation index as compared with the wild-type cells and the appearance of multi-septated phenotypes. More importantly, both the mutant alleles showed similar defects in membrane trafficking, including defects in secretion and Golgi/endosomal trafficking as evidenced by the mislocalization of the synaptobrevin Syb1 and the Golgi marker protein Vrg4 (Figure 4). Furthermore, both the mutants did not show defects in the internalization step of endocytosis, although some FM4-64 fluorescent dots were observed in most of the *sip1-i4* and *sip1-62* mutants (Figure 5). Jourdain *et al.* reported mislocalization of FM4-64 in the *sip1-62* mutants. In normal cells, FM4-64 is delivered to the vacuole. In the mutants even at a permissive temperature FM4-64 is localized at the tips and mid-zone of the cell. Because our data clearly showed transport of Lucifer yellow to the vacuoles in the *sip1-62* mutants, we concluded that Sip1 was actually involved in post-endocytic endosomal trafficking toward vacuoles and not in endocytosis. We also observed a genetic interaction between Sip1 and calcineurin. Because the *its4-1*/*sip1-i4* allele showed sensitivity to the calcineurin inhibitor FK506, it may be predicted that Sip1 and calcineurin share an essential function for growth. A possible mechanism of the synthetic lethal interaction between *sip1-i4* and calcineurin is cell wall integrity defect. Notably, Bgs1 was localized at Golgi/endosomes in addition to its reported localization at the cell surface and septa, and these localizations were dependent on the Sip1/AP-1 complex. Therefore, the combination of the *sip1* mutation and the inhibition of calcineurin by FK506, through mislocalization and the decreased gene expression of Bgs1, may cause severe Bgs1 dysfunction and cell wall defects, which can explain the synthetic lethal interaction. In budding yeast, Valdivia *et al.* reported such a role for AP-1 in the correct sorting of the chitin synthase Chs6 [39]. However, to our knowledge, there has been no report that a glucan synthase represents a Golgi/endosome localized membrane protein acting as a cargo for the Sip1/AP-1 complex.

We have provided evidence that the Sip1/AP-1 complex maintains the integrity of Golgi/endosomal trafficking, mediates transport of Bgs1. Identifying additional immunosuppressant sensitive mutants may reveal novel players whose functions are important for Golgi/endosomal trafficking.

## Supporting Information

Figure S1 **C-terminal tagging of Sip1 by GFP does not affect Sip1-GFP function, and C- or N-terminal GFP-tagged Sip1 partially co-localizes with Sec72-mCherry.** (A) C-terminally tagged Sip1-GFP strain exhibited no sensitivity to high temperature, FK506, or micafungin. Wild-type cell (wt), *sip1-i4* mutant cells (*sip1-i4*), and Wild-type (wt) cells that expressed chromosome-borne Sip1-GFP were streaked onto plates containing YES or YES plus FK506 (0.5 µg/mL), micafungin (0.5 µg/mL), followed by incubation at 27°C for 4 d or at 36°C for 3 d. (B) Sip1-GFP or GFP-Sip1 did not co-localize with the Golgi marker Anp1-mCherry (*cis*-Golgi) in wild-type cells. Wild-type cells expressed chromosome-borne Anp1-mCherry and Sip1-GFP or chromosome-borne Anp1-mCherry and GFP-Sip1 under the control of the *nmt1* promoter. Cells were cultured in YPD medium at 27°C and examined by fluorescence microscopy. (C) Partial co-localization of Sip1-GFP or GFP-Sip1 with the Golgi marker Sec72-mCherry (*trans*-Golgi) in wild-type cells. Wild-type cells expressed chromosome-borne Sec72-mCherry and Sip1-GFP, or chromosome-borne sec72-mCherry and GFP-Sip1 under the control of the *nmt1* promoter. Cells were cultured and observed as described in B. Arrowheads indicate the co-localization of Sip1-GFP or GFP-Sip1 with Sec72-mCherry at *trans*-Golgi. Bar, 10 µm.
(TIF)

Figure S2 **Subcellular localizations of Sip1-GFP in subunit deletion cells are similar to that in Wild-type cells.** (A) Subcellular localizations of Sip1-GFP in wild-type (wt), Apm1-deletion cells (Δ*apm1*), Apl2-deletion cells (Δ*apl2*), Apl4-deletion cells (Δ*apl4*), and Aps1-deletion cells (Δ*aps1*). Cells that expressed chromosome-borne Sip1-GFP were cultured in YPD medium at 27°C. They were incubated with FM4-64 dye for 5 min at 27°C to visualize Golgi/endosomes. Arrowheads indicate the localization of Sip1-GFP at Golgi/endosomes. Bar, 10 µm. (B) Sip1-GFP did not co-localize with the Golgi marker Anp1-mCherry (*cis*-Golgi) in Apm1-deletion cells (Δ*apm1*). Apm1-deletion cells expressed chromosome-borne Anp1-mCherry and Sip1-GFP. Cells were cultured in YPD medium at 27°C and examined by fluorescence microscopy. (C) Partial co-localization of Sip1-GFP with the Golgi marker Sec72-mCherry (*trans*-Golgi) in Apm1-deletion cells (Δ*apm1*). Apm1-deletion cells expressed chromosome-borne Sec72-mCherry and Sip1-GFP. Cells were cultured and observed as described in B. Arrowheads indicate the co-localization of Sip1-GFP with Sec72-mCherry at *trans*-Golgi. Bar, 10 µm. Cells were cultured and observed as described in B.
(TIF)
