# Supplementary figures and images for: Sip1, a Conserved AP-1 Accessory Protein, Is Important for Golgi/Endosome Trafficking in Fission Yeast
Source: PLoS One. 2012 Sep 17;7(9):e45324. doi: 10.1371/journal.pone.0045324 (PMC3444471; doi:10.1371/journal.pone.0045324)

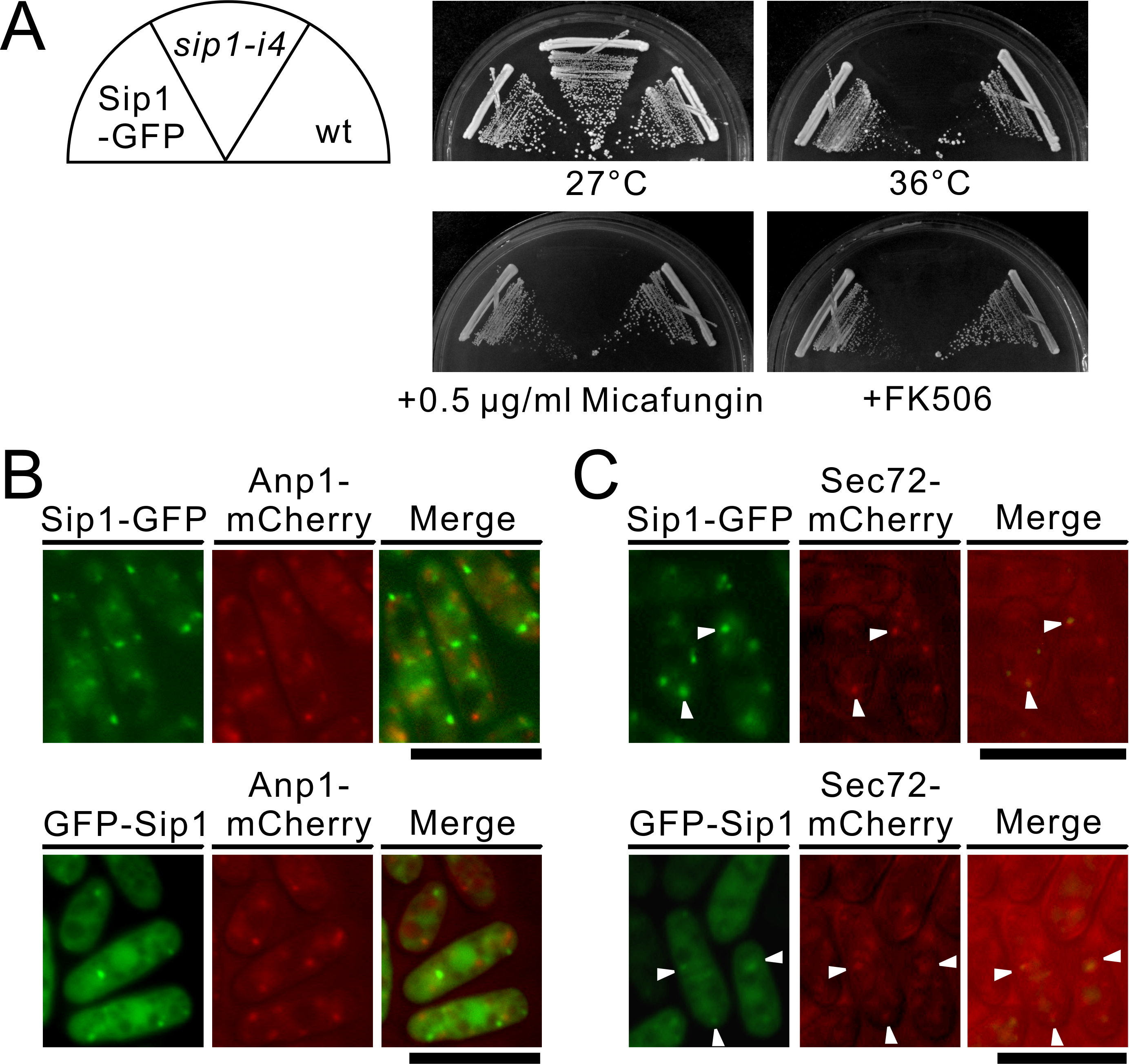

Supplement: Figure S1 — C-terminal tagging of Sip1 by GFP does not affect Sip1-GFP function, and C- or N-terminal GFP-tagged Sip1 partially co-localizes with Sec72-mCherry. (A) C-terminally tagged Sip1-GFP strain exhibited no sensitivity to high temperature, FK506, or micafungin. Wild-type cell (wt), sip1-i4 mutant cells (sip1-i4), and Wild-type (wt) cells that expressed chromosome-borne Sip1-GFP were streaked onto plates containing YES or YES plus FK506 (0.5 µg/mL), micafungin (0.5 µg/mL), followed by incubation at 27°C for 4 d or at 36°C for 3 d. (B) Sip1-GFP or GFP-Sip1 did not co-localize with the Golgi marker Anp1-mCherry (cis-Golgi) in wild-type cells. Wild-type cells expressed chromosome-borne Anp1-mCherry and Sip1-GFP or chromosome-borne Anp1-mCherry and GFP-Sip1 under the control of the nmt1 promoter. Cells were cultured in YPD medium at 27°C and examined by fluorescence microscopy. (C) Partial co-localization of Sip1-GFP or GFP-Sip1 with the Golgi marker Sec72-mCherry (trans-Golgi) in wild-type cells. Wild-type cells expressed chromosome-borne Sec72-mCherry and Sip1-GFP, or chromosome-borne sec72-mCherry and GFP-Sip1 under the control of the nmt1 promoter. Cells were cultured and observed as described in B. Arrowheads indicate the co-localization of Sip1-GFP or GFP-Sip1 with Sec72-mCherry at trans-Golgi. Bar, 10 µm. (TIF) [file pone.0045324.s001.tif]

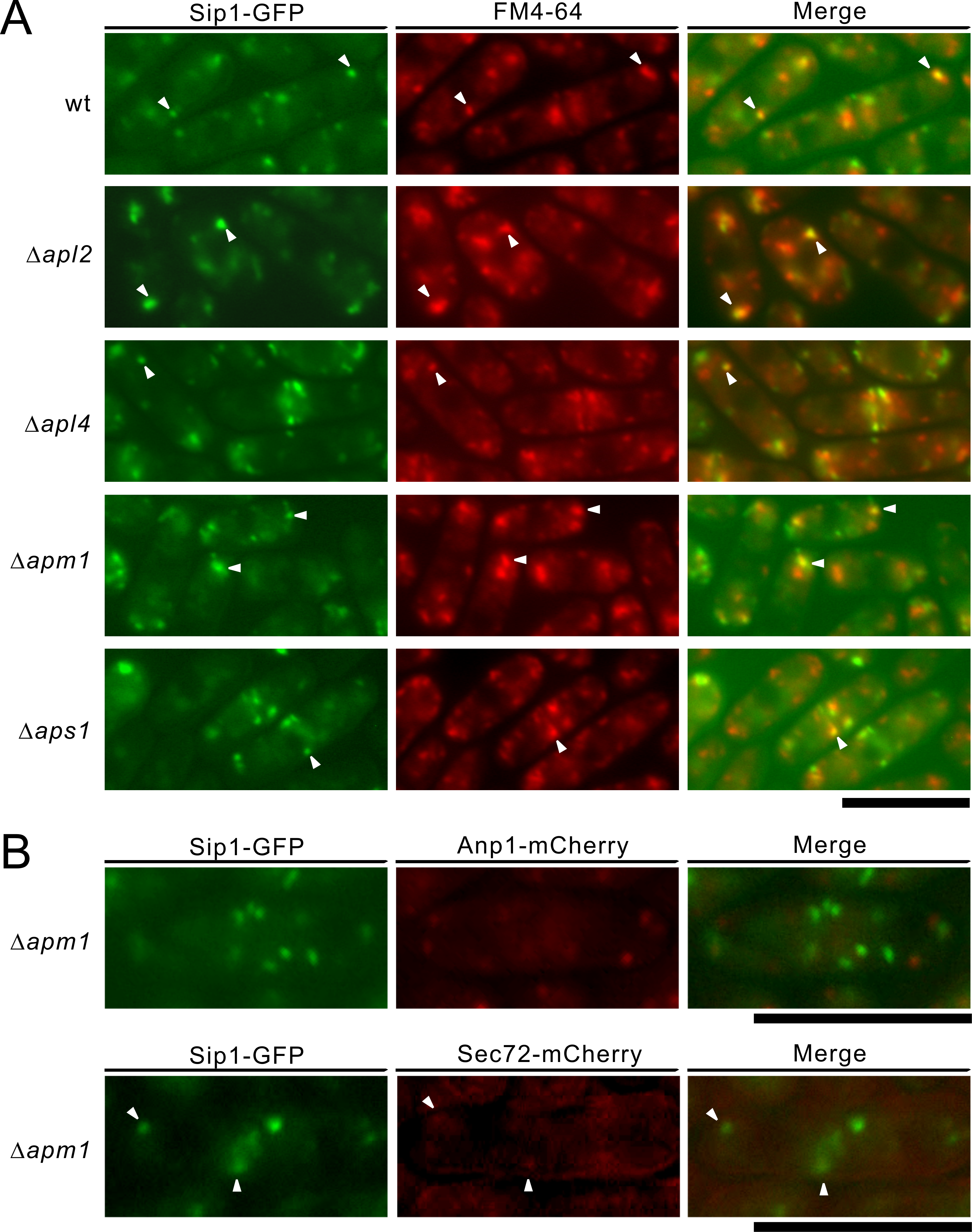

Supplement: Figure S2 — Subcellular localizations of Sip1-GFP in subunit deletion cells are similar to that in Wild-type cells. (A) Subcellular localizations of Sip1-GFP in wild-type (wt), Apm1-deletion cells (Δapm1), Apl2-deletion cells (Δapl2), Apl4-deletion cells (Δapl4), and Aps1-deletion cells (Δaps1). Cells that expressed chromosome-borne Sip1-GFP were cultured in YPD medium at 27°C. They were incubated with FM4-64 dye for 5 min at 27°C to visualize Golgi/endosomes. Arrowheads indicate the localization of Sip1-GFP at Golgi/endosomes. Bar, 10 µm. (B) Sip1-GFP did not co-localize with the Golgi marker Anp1-mCherry (cis-Golgi) in Apm1-deletion cells (Δapm1). Apm1-deletion cells expressed chromosome-borne Anp1-mCherry and Sip1-GFP. Cells were cultured in YPD medium at 27°C and examined by fluorescence microscopy. (C) Partial co-localization of Sip1-GFP with the Golgi marker Sec72-mCherry (trans-Golgi) in Apm1-deletion cells (Δapm1). Apm1-deletion cells expressed chromosome-borne Sec72-mCherry and Sip1-GFP. Cells were cultured and observed as described in B. Arrowheads indicate the co-localization of Sip1-GFP with Sec72-mCherry at trans-Golgi. Bar, 10 µm. Cells were cultured and observed as described in B. (TIF) [file pone.0045324.s002.tif]
